# Emergence of task-dependent representations in working memory circuits

**DOI:** 10.3389/fncom.2014.00057

**Published:** 2014-05-28

**Authors:** Cristina Savin, Jochen Triesch

**Affiliations:** ^1^Frankfurt Institute for Advanced StudiesFrankfurt am Main, Germany; ^2^Physics Department, Goethe UniversityFrankfurt am Main, Germany

**Keywords:** working memory, reward-dependent learning, STDP, intrinsic plasticity, synaptic scaling, prefrontal cortex, delayed categorization

## Abstract

A wealth of experimental evidence suggests that working memory circuits preferentially represent information that is behaviorally relevant. Still, we are missing a mechanistic account of how these representations come about. Here we provide a simple explanation for a range of experimental findings, in light of prefrontal circuits adapting to task constraints by reward-dependent learning. In particular, we model a neural network shaped by reward-modulated spike-timing dependent plasticity (r-STDP) and homeostatic plasticity (intrinsic excitability and synaptic scaling). We show that the experimentally-observed neural representations naturally emerge in an initially unstructured circuit as it learns to solve several working memory tasks. These results point to a critical, and previously unappreciated, role for reward-dependent learning in shaping prefrontal cortex activity.

## 1. Introduction

Working memory is defined as the temporary storage of stimulus-specific information during a delay period. This function has been traditionally associated with circuits in prefrontal cortex (PFC). Classic work in monkeys revealed that single neurons in this region exhibit selective persistent activity during the delay period (Miyashita, 1988; Goldman-Rakic, 1990) and its disruption (by electrical stimulation, or due to distracters) leads to a decay in performance (Funahashi et al., 1989). These early observations have been interpreted as the circuit exhibiting attractor dynamics, which enable a subset of the neurons to maintain high firing throughout the delay after a brief stimulus presentation (Amit and Brunel, 1997; Brunel and Wang, 2001). This view has been revised in recent years, as it was shown that most neurons in PFC change their firing rates during the delay (Miller et al., 1996; Chafee and Goldman-Rakic, 1998; Pesaran et al., 2002; Rainer and Miller, 2002; Barak et al., 2010), suggesting that working memory circuits rely on feedforward rather than attractor dynamics (Goldman, 2009). Still, while experiments generally agree on how information is represented in working memory circuits, i.e., using spatio-temporal patterns of neural activity, exactly what information gets encoded is less clear.

An accumulation of data across different working memory experiments paints an increasingly complex picture of the features encoded in PFC. We find neurons may represent the previous stimulus, the forthcoming action, or a more complex function of the two (Durstewitz et al., 2000). When the task requires a generalization across stimuli, neurons develop category selectivity (Freedman et al., 2001). Moreover, there is a gradual shift in these representations as the number of examples per class increases, with animals switching from a stimulus-response association strategy to representing categorical distinctions directly (Antzoulatos and Miller, 2011).

Things get even more complicated when animals need to alternate between different tasks. While PFC neurons generally represent the task to be performed (Asaad et al., 1998; Cromer et al., 2010; Roy et al., 2010; Warden and Miller, 2010; Meyer et al., 2011), they can differ significantly with respect to how the information is distributed across the population in different tasks. For instance, neurons can show task-specific changes in overall firing rate, in time-dependent response profiles and in stimulus and response selectivity (Asaad et al., 2000). In some situations, the same neurons seem to participate in encoding features related to different tasks (e.g., making different category distinctions, Cromer et al., 2010), effectively multiplexing information across contexts. In other situations, however, information is encoded in different neurons for different contexts (Roy et al., 2010), and—worse still—it is unclear when one or the other coding strategy may be employed. Generally, we are missing a unifying account for PFC representations during the delay period.

Here we hypothesize that reward-dependent learning underlies the variety in PFC representations in different working memory tasks. The data itself suggest that this may be the case: the most striking feature of the above experiments is not the diversity of neural responses, but the sheer number of neurons displaying an effect. Regardless of the actual task the monkey has been trained to carry out, a significant subset of the recorded neurons are found to exhibit selectivity to the specifics of that particular task. This is a strong indication that PFC neurons adapt their responses to reflect current cognitive demands. Indeed, PFC representations change significantly over the course of training (Rainer et al., 1998b; Rainer and Miller, 2000; Baeg et al., 2003; Kennerley and Wallis, 2009). Neural responses become increasingly sparse, the tuning of the neurons narrows, and the representation becomes more robust to input noise (Rainer and Miller, 2000). These changes in neural representation parallel behavioral learning, and allow for a better decoding of stimuli and actions (Baeg et al., 2003). Moreover, since the training-induced changes in neural responses include changes in functional connectivity (Baeg et al., 2007), it seems likely that associative learning within the circuit is responsible—at least in part—for the refinement of neural representations with learning. The specific mechanisms involved remain unclear, however.

We assume that learning in PFC is reward-dependent. This hypothesis is consistent with the observation that dopamine, a neuromodulator associated with reward prediction error (Schultz, 1998), modulates synaptic plasticity in this circuit (Otani et al., 2003). It also explains the dependence of neural representations on the magnitude of the expected reward (Kennerley and Wallis, 2009). However, the primary reason for our assumption is computational. Working memory circuits are know to operate under strict capacity constraints (Cowan, 2001), and a circuit with limited resources cannot simply encode everything. To perform well, it needs to represent the specific aspects of the stimulus that matter for the task at hand (Duncan, 2001). Hence, reward should modulate learning so as to shift representations toward task-relevant features. This points to a critical and previously unrecognized role for reward-dependent plasticity in shaping prefrontal representations.

Can reward-dependent learning alone explain the wide variety of experimental observations on PFC encoding? To address this question, we studied the effects of reward-dependent learning on the encoding properties of neurons in a working memory circuit. More specifically, we trained a generic recurrent neural network to solve tasks similar to those employed in working memory experiments. We then investigated the neural representations emerging in the circuit and compared them to neural data. We chose a simple abstract model for the network dynamics in which the output of a neuron depends only on its instantaneous inputs. Learning was implemented by reward-dependent spike timing dependent plasticity (rSTDP) (Izhikevich, 2007), supplemented by homeostatic mechanisms that stabilized the network dynamics during learning (Lazar et al., 2009). Importantly, as individual neurons have no memory themselves, the storage of information in this circuit relies exclusively on the recurrent connectivity. While this simple model cannot capture the full complexity of the temporal dynamics in PFC, it allows us to focus specifically on the reward-dependent reorganization of recurrent connections and its effects on circuit function.

We found that our model is able to capture key aspects of neuronal dynamics during working memory tasks. Neurons in the model develop specificity in space and time and, depending on the task, they preferentially encode individual stimuli, actions, or context information. In a simple delayed-response task, neurons encode stimulus identity (Miller et al., 1996; Constantinidis and Franowicz, 2001). In a delayed-categorization task, neurons learn to preferentially encode category boundaries (Freedman et al., 2001). Lastly, when learning several tasks at the same time, the degree of neural specialization depends on the specifics of the task, mirroring experimental data. When the task involves several independent category schemes, neurons act as “multiplexers,” coding for different things in different contexts (Cromer et al., 2010); when the same stimuli need to be categorized differently depending on behavioral context, the neurons segregate into distinct task-specific subpopulations (Roy et al., 2010). Furthermore, reward-dependent learning is critical for these results. A similar circuit trained by unsupervised learning shows a significant loss in working memory performance, paired with poorer neural representations. Taken together, our findings show reward-dependent learning could be a central force in the organization of working memory circuits.

## 2. Materials and methods

### 2.1. The general task

**Figure 1:**
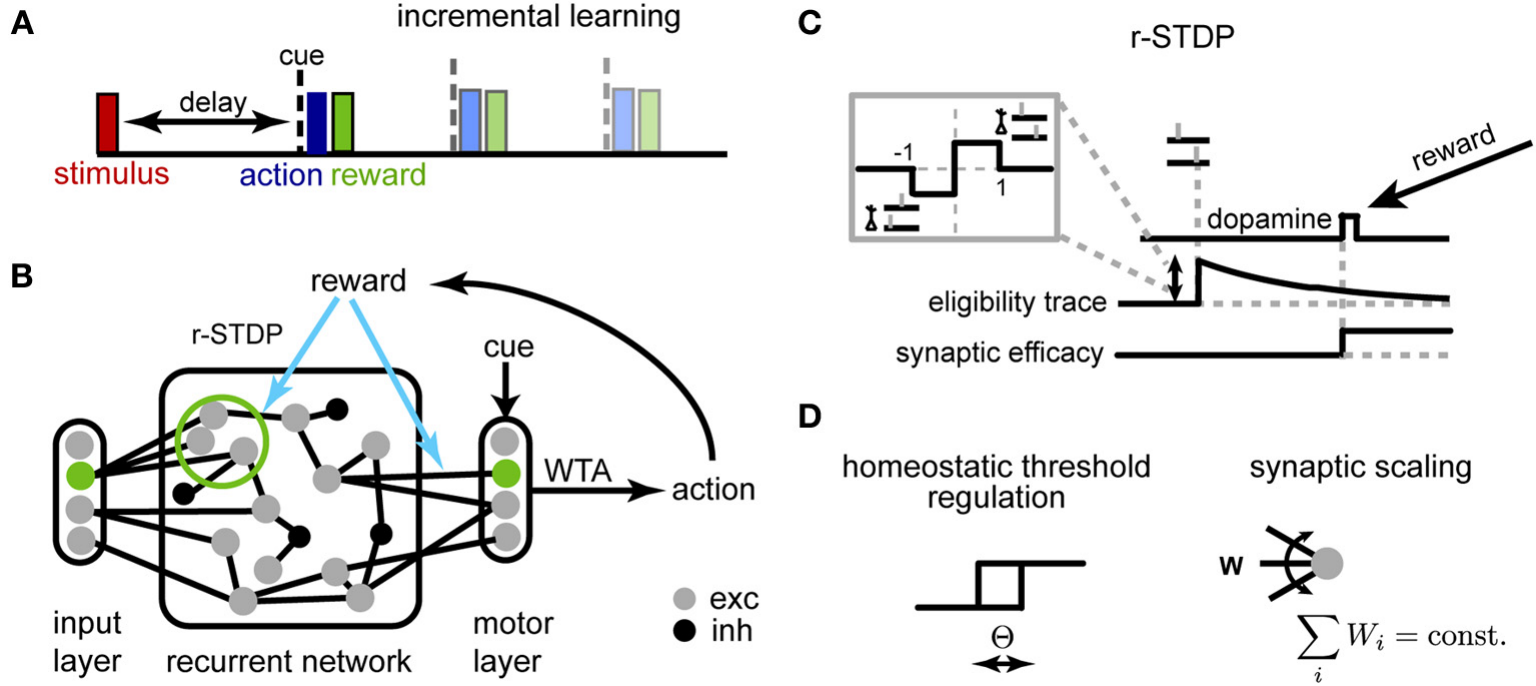
**Schematic description of the model. (A)** Delayed response task: at the beginning of each trial, one of *K* stimuli is presented to the network, requiring a stimulus-dependent action to be performed at the end of the delay period. When the cue appears, an action is selected yielding a corresponding reward. Initial trials have short delays, and we progressively increase the delay period during learning. **(B)** The network of threshold linear neurons receives localized, stimulus-specific inputs; the decision units determine the action to be performed by winner-take-all (WTA). The corresponding reward modulates plasticity events at synapses within the recurrent network and to the decision units. **(C)** Reward-dependent STDP is implemented using eligibility traces, with changes occurring only at the time of reward (see main text for details); additionally, **(D)** the neuron threshold is homeostatically regulated and the incoming synapses to each neuron are normalized.

The working memory tasks we investigated share a simple general structure (Figure 1A): at the beginning of a trial one stimulus (out of *K*) is briefly presented to the network. After a delay period (either fixed for a block of trials or selected at random from a given distribution) a “Go” cue is presented, after which the reward is given according to the action selected by the model (one out of *M*)—either +1 for a correct answer or −1, otherwise. Different tasks correspond to different mappings between stimuli and actions and each are described in detail in the corresponding Results section. To speed up learning, we adopt the same strategy employed in training animals for experiments, i.e., we start with the minimum delay version of the task and progressively increase the duration of the delay period during learning (Klingberg, 2010).

### 2.2. Network model

An overview of the network is shown in Figure 1B. The recurrent network consists of *N* units (unless otherwise specified, *N* = 250), 80% excitatory and 20% inhibitory, with sparse random connectivity. Input units encoding different stimuli (and possibly the context cue) activate small, non-overlapping subsets within the recurrent layer, each consisting of *N*_in_ excitatory neurons *N*_in_ = 5; the activation of the input unit provides a suprathreshold current which forces the corresponding subpopulation to be active for one time step. The output layer receives inputs from all excitatory units within the network and generates a decision response through a winner-take-all (WTA) mechanism. This decision outcome determines the received reward, which in turn modulates synaptic changes through r-STDP. Reward-dependent learning affects both excitatory synapses within the recurrent network and those connecting to the decision layer.

We chose a abstract model for the neural dynamics, whose simplicity allows us to focus on the essential mechanisms required for explaining the data. More specifically, we used linear threshold units to model neurons within the network, i.e., each unit has a binary output:

(1) $$x_{i}(t)=I_{i}(t)\geq \Theta_{i},$$

with activation depending on the total current to the neuron *I*_*i*_(*t*) and the neuron's spike threshold Θ_*i*_ (this threshold also changes over a slower time scale because of homeostatic mechanisms, see below). The activity proceeds in discrete time steps, with synchronous updates for all neurons. The input to a neuron is given by:

(2) $$I_{i}=w_{i}^{T}\cdot \text{x}+\epsilon ,$$

where column vectors w and x describe the synaptic weights and the activity of all presynaptic neurons, respectively. The stochastic term ϵ corresponds to an unspecific background input to each unit, modeled as independent uniform random noise, ϵ ∈ [0, 0.1]. Importantly, since the model neuron has no memory itself, working memory can develop in the model only through the network dynamics. Hence, we can use the model to study specifically reward-dependent plasticity and its effects on information storage.

The connectivity matrix was initialized randomly at the beginning of each experiment, with weights drawn from the uniform distribution *w*_*ij*_ ∈ [0, 1], followed by a sum-to-one weight normalization of incoming synapses. The connection probabilities were *p*_*ee*_ = 0.1, *p*_*ei*_ = 0.25, *p*_*ie*_ = 0.4, *p*_*ii*_ = 0, with indices “e” and “i” marking the excitatory and inhibitory populations, respectively.

For the decision layer, the current to each neuron is computed as before, with the WTA mechanism selecting the neuron with the strongest input as the only active unit: *I*_*m*_ = *w*^*T*^_*i*_ · *x* + ϵ, *x*_*m*_ = 1 if *m* = argmax_*j*_ *I*_*j*_, and *x*_*m*_ = 0, otherwise. Decision neurons were allowed to fire during the delay period without any effect on reward.

### 2.3. Plasticity mechanisms

#### 2.3.1. Reward-dependent learning

We adapted a model for r-STDP from Izhikevich (2007) (Figure 1C). As in the original, each synapse has an associated eligibility trace *e*_*ij*_:

(3) $$\frac{de_{ij}}{dt}=-\frac{e_{ij}}{\tau_{\text{e}}}+x_{i}(t)\cdot x_{j}(t-1)-f\cdot x_{i}(t-1)\cdot x_{j}(t)$$

where *x*_*i*_ and *x*_*j*_ are the output of the pre- and post-synaptic neuron, respectively, and *f* is a model parameter (*f* = 1 for synapses in the recurrent layer, and *f* = 0.01 for synapses in the motor layer).

The eligibility trace stores a history of potential weight changes at the synapse, with an exponential decay, specified by the time constant τ_e_ (τ_e_ = 2.5). The individual synaptic plasticity events follow a simplified STDP window: potentiation occurs when presynaptic activity is followed by a postsynaptic spike, while the reverse pattern causes depression, with a width of 1 time step (since that is the timescale of causal interactions in our network). Additionally, weights are rectified such that *w*_*ij*_ ≥ 0 in order to respect Dale's law.

At the time of the reward synaptic weights change proportionally to the eligibility trace *e*_*ij*_ and the reward signal *r*:

(4) $$w_{ij}(t+1)=w_{ij}(t)+\eta \cdot r(t)\cdot e_{ij}(t),$$

with learning rate η.

For simplicity, we used the absolute reward as the signal modulating synaptic modifications instead of the reward prediction error (Schultz, 1998), as done in previous models (Izhikevich, 2007). Additionally, we assumed the reward to be either positive or negative, as biological evidence from cortico-striatal synapses suggests that dopamine can induce both potentiation or depression in response to tetanic stimulation, depending on its concentration relative to baseline (Reynolds and Wickens, 2002). Specifically, at the time of the reward delivery *r*(*t*) = 1, if the motor output was correct and *r*(*t*) = −1, otherwise; *r*(*t*) = 0 at all other times.

To ensure that the system is given time to exploit the emerging neural representation, we assumed that changes at synapses to the decision layer occur faster than those in the recurrent network (η = 10^−5^ for synapses in the recurrent layer and η = 10^−4^ for those connecting to decision neurons). These changes in learning rate were paralleled for intrinsic plasticity to ensure that the dynamics remain stable during learning (see below).

#### 2.3.2. Homeostatic plasticity

A critical problem when optimizing recurrent networks is how to stabilize the dynamics during learning (Turrigiano and Nelson, 2004; Lazar et al., 2009). Traditionally, working memory models with attractor dynamics circumvent this problem by keeping weights fixed and fine-tuning a limit set of gain parameters by hand (Brunel and Wang, 2001). Here, we use two distinct homeostatic mechanisms to ensure stability (Figure 1D): synaptic scaling (Turrigiano et al., 1998) and homeostatic threshold regulation (Zhang and Linden, 2003).

First, as synaptic scaling constrains the total drive received by neurons by rescaling all weights in a multiplicative fashion, we implemented this mechanism by an explicit weight normalization, Σ_*j*_*w*_*ij*_ = 1. We chose this for simplicity, although a similar outcome could in principle be achieved through a local weight-dependent rule (Gerstner and Kistler, 2002). Second, intrinsic plasticity was implemented by assuming that the threshold of excitatory neurons adapts to maintain a certain mean average firing rate, *x*_0_ ∈ (0, 1):

(5) $$\Delta \Theta =\lambda_{\text{exc}}\left(x(t)-x_{0}\right)\text{​},$$

where λ_exc_ is the time constant for the threshold adaptation (*x*_0_ = 0.03 within the recurrent network and *x*_0_ = 0.25 in the decision layer). As mentioned above, the timescale of plasticity for the decision units is 10 times faster to match the more rapid synaptic plasticity (λ_exc_ = 10^−4^ within the recurrent layer, and λ_exc_ = 10^−3^ for the decision units).

We assumed a similar threshold regulation for controlling the excitability of the inhibitory neurons. The specific form was suggested by experimental evidence showing that the excitability of inhibitory neurons is determined by the overall activity of neighboring excitatory neurons, estimated via the release of diffusible messengers, such as BDNF (Rutherford et al., 1998; Turrigiano and Nelson, 2000). Specifically, we assume that the threshold of inhibitory neurons changes as:

(6) $$\Delta \Theta_{\text{inh}}=-\lambda_{\text{inh}}\left(\langle x_{\text{exc}}(t)\rangle -x_{0}\right)\text{​},$$

with 〈*x*_exc_(*t*)〉 denoting the population average of the activation of all excitatory neurons at time *t*. This is a simplification of a more realistic input-specific regulation of excitability chosen for convenience, consistent with inhibitory neurons pooling activity across a large part of the circuit. As before, *x*_0_ is the desired average firing rate of the excitatory neurons, and λ_inh_ is the learning rate (λ_inh_ = 10^−5^). Although this mechanism is not strictly necessary for network stability, we find it improves memory performance and ensures a fairer distribution of neuronal resources across stimuli.

#### 2.3.3. Other simulation parameters

All trials are assumed to have fixed duration *T*_trial_ = 10 time steps, with 2 · 10^4^ trials per block. We repeat each experiment five times to quantify effects different sources of variability, such as the network initialization, internal noise, etc.

## 3. Results

### 3.1. A delayed response task

The most common experimental paradigm for exploring the circuits involved in working memory is the delayed response task, where a simple stimulus-specific response needs to be delivered after a delay (Rainer et al., 1998a; Durstewitz et al., 2000). Computational models of this function assume a circuit with distinct submodules for storing the initial stimulus (the working memory component), comparing it to the sample and deciding on the action (Engel and Wang, 2011). Here we focus on the first component, and thus assume a one-to-one mapping between stimuli and actions (*M* = *K*). Although we neglect the intermediate step, i.e., making same-or-different judgements, nonetheless the model preserves the nature of the underlying computation. Hence this simplification should not affect our results concerning the representation within the working memory circuit.

**Figure 2:**
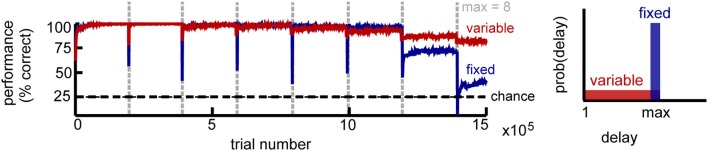
**Circuit performance for a simple delayed response task**. Recall performance as a function of the number of trials for *K* = 4 stimuli. Vertical lines mark the time when the maximum delay is incremented. Within a block the maximum delay is kept fixed, with two variations: in the fixed delay task (blue) all trials have the same delay, while in the variable delay task (red) the delay of each trial is drawn independently from the uniform distribution [1, max]. Performance estimated across 100 trial blocks.

We used two variants for the basic setup: a fixed-and a variable-delay version (Figure 2, right). As its name suggests, the first uses a fixed delay for all trials in a block. This version is useful for estimating the memory capacity of the network, defined as the longest delay for which performance is better than chance. However, it could potentially lead to unrealistic delay-specific representations. In the second setup, the delay for each trial is selected uniformly at random between one and a maximum delay *T*_max_ time steps. This version seems closer to the true constraints of the biological system, where information needs to be accessible on demand whenever the the environmental conditions call for it. Hence, we used the second version of the task to investigate the emerging neural representations.

We found that the network performance is influenced by task difficulty (Figure 2). As expected, it decreases with increasing delay, due to the accumulation of noise. For intermediate delays, the fixed-delay task yields slightly better results compared to the variable-delay task, consistent with it being computationally simpler. At longer delays however, the network exhibits a sharp performance decay, which signals the network reaching its memory capacity. In the variable delay task, performance degrades more gracefully, as shorter memory spans are still rewarded. In both cases, we found that recall performance increases with network size *N*, and decays with the number of distinct stimuli *K* and that the incremental learning paradigm dramatically improves network performance (not shown).

Importantly, performance is remarkably stable within a block of trials despite the constant changes induced by the different plasticity mechanisms, with the network reaching the final performance after a small number of trials (on the order of 100 trials). The critical condition to achieve such good and stable performance is a sparse representation within the recurrent layer (enforced by intrinsic plasticity), combined with balanced rSTDP. While not strictly necessary, synaptic scaling and inhibitory plasticity improve performance; additionally we found it was beneficial to reduce the LTD component for learning in the motor layer (presumably because it limits the interference due to motor activity in the delay period). Overall, the interaction between different plasticity mechanisms is needed for the circuit to maintain stable function despite variable underlying neural “hardware.”

**Figure 3:**
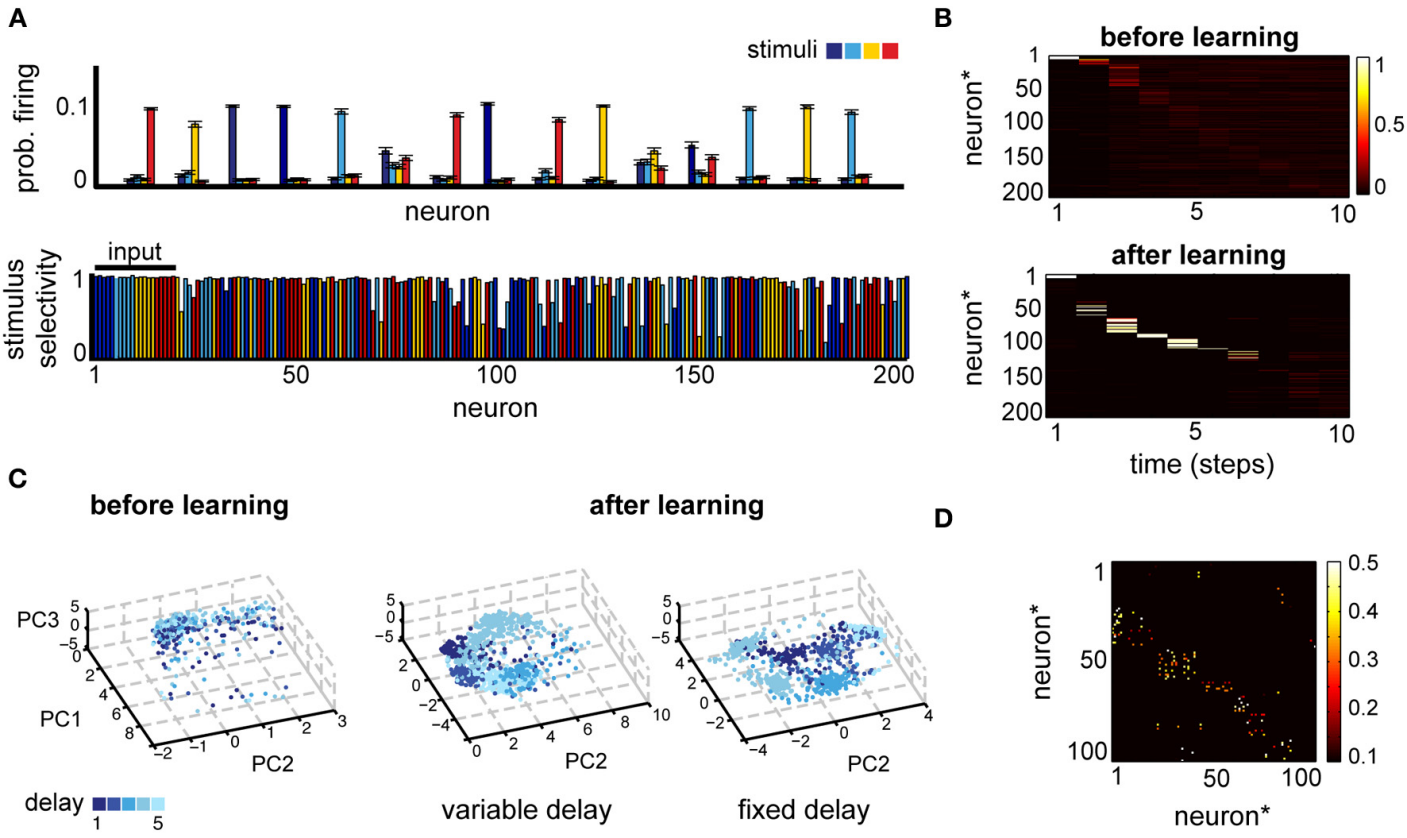
**(A)** Neural selectivity in a simple delayed response task (*T*_max_ = 5, variable delay). Top: neural responses averaged across trials where one of four stimuli (different colors) was presented, for a subset of 15 randomly selected neurons. Bottom: selectivity of neural responses across the population for one example experiment; estimated using activity in 1000 trials at the end of learning. Note that the first 20 neurons receive direct inputs from the input layer. **(B)** Comparison of the post-stimulus time histogram of neural responses before and after learning for one example stimulus. Neuron indices have been reordered based on the time of maximum firing relative to stimulus onset. **(C)** A low-dimensional view of the population dynamics in response to the same stimulus before learning (left) or after training using either the fixed (right) or the variable delay (middle) paradigm. Individual points correspond to the state of the network projected along the first three principal components; color intensity marks the time since the stimulus presentation and different points of the same color correspond to different trials. **(D)** The corresponding weight matrix at the end of leaning.

To examine the representation that emerges after learning, we measured both the spatial and the temporal selectivity of neural responses. For the spatial component, we computed the average neural activation during the delay period for each stimulus (Figure 3A, top). This simple measure reveals that most of the neurons respond to one of the stimuli, while remaining relatively silent for the others, as demonstrated in classic working memory experiments (Miyashita, 1988). To better quantify the effect, we used a measure called the depth of selectivity (Rainer et al., 1998b), defined as:

(7) $$S=\frac{N_{\text{cond}}-\frac{\Sigma R_{i}}{R_{\text{max}}}}{N_{\text{cond}}-1},$$

where *R*_i_ is the firing rate corresponding to stimulus *i*, *R*_max_ = max{*R*_i_} and *N*_cond_ is the number of different behavioral states considered, here the *K* stimuli. This measure takes the value zero when the neural response is identical for all objects and can reach the maximum of one when the neuron responds exclusively to one of the stimuli. Note that we will use this measure more generally in the following sections, to also measure the specificity to distinct actions or contexts. The depth of selectivity confirmed that most neurons exhibit stimulus-dependent activation (Figure 3A, bottom).

Neural responses are structured also in the temporal domain, reproducing at least qualitatively the temporal specificity in experiments (Meyers et al., 2008). A post-stimulus time histogram (PSTH) of the network responses for a given stimulus reveals that, although before learning the response is highly variable (Figure 3B, top and Figure 3C, left), after learning neuronal responses become highly reproducible (Figure 3B; note that neuron indices were reordered as a result of sorting the neuron by the time of the peak response). Moreover, neurons respond at specific times relative to stimulus onset, pointing to a synfire chain-like representation (Aertsen et al., 1996; Prut et al., 1998). Such temporal dynamics allow neurons to remain stimulus specific, while maintaining a sparse activation enforced thorough the homeostatic regulation of neural excitability. Additionally, the network dynamics reflect the details of the task (Figure 3C): the delay itself is encoded much better during the fixed-delay version of the task. A low-dimensional projection of the neural activity by principal component analysis (PCA) reveals distinct vs. overlapping stimulus-specific clusters in the fixed- and the variable delay task, respectively. This reflects the intuition that the time since the stimulus presentation is important for the fixed delay task, whereas in the variable delay version the motor layer just needs to linearly separate the activity corresponding to different stimuli, irrespective of the delay. The time-dependent encoding is also reflected in the connectivity matrix, which becomes sparse and more feedforward (Figure 3D). More generally, learning organizes the network in largely non-overlapping feedforward chains, each starting from one of the input sub-populations and with a total size determined by the number of inputs, the size of the network, and the sparseness enforced through the homeostatic mechanisms (not shown). In summary, in a simple delayed-response task, the network uses distributed representations for encoding information about the stimuli across time and space, in a way that makes it easily accessible for decision circuits and is consistent with experiments.

### 3.2. A delayed categorization task

Neurons in PFC can encode either the initial stimulus, or the action to be taken in response to it (Brody et al., 2003). For the simple delayed-response task above there is no difference between the two, as actions simply signal stimulus identity. To investigate under which conditions the circuit learns to represent preferentially stimuli or actions, we used a delayed categorization task, inspired by experiments in monkeys, in which arbitrary categories are defined using morphed images (generated from e.g., cat and dog prototypes), see Freedman et al. (2001).

**Figure 4:**
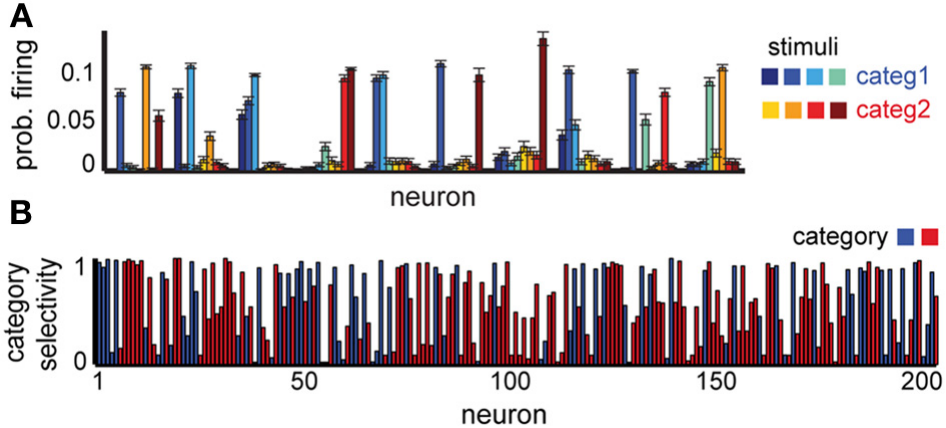
**Neural selectivity in a delayed categorization task. (A)** Average responses to each stimulus in 10 randomly selected example neurons. **(B)** Specificity of neural responses to one of the two categories (red or blue); colors show preferred category for each neuron. Neural selectivity was estimated using activity in 1000 trials at the end of learning. *T*_max_ = 5, variable delay.

To mimic this paradigm, we constructed an arbitrary map between *K* = 8 stimuli and *M* = 2 decision outputs signaling stimulus class. Here, category boundaries are defined exclusively by the reward function (Freedman et al., 2001; Antzoulatos and Miller, 2011), unlike some experiments in which category specificity may be—to some extent—stimulus driven (Meyers et al., 2008). For illustration purposes, we define the mapping by stimulus color (Figure 4A, right), though in the model the random initialization of the connectivity makes any subdivision of non-overlapping stimuli to be equivalent.

Our network is able to successfully learn the task (75% correct for a delay of five time steps). The neural representations for this task show some novel characteristics compared to the simple delay task, which reflect the experimental data (Freedman et al., 2001). While some of the neurons still respond selectively to individual stimuli, a significant subpopulation responds now to several stimuli, and often to those belonging to the same category (Figure 4A). Using the depth of selectivity (with categories rather than stimuli as behaviorally relevant variable) enables us to quantify the category selectivity of neurons across the population (Figure 4B). Using this metric, we found that a significant fraction of the neurons (32% of excitatory neurons have *S* ≥ 0.75) exhibit category selectivity, close to the 33% reported in monkeys (Freedman et al., 2001). As in the previous experiment, their representations are time-varying; at any time, only a small fraction of neurons encode category information, with information being passed between different small subsets of neurons over the course of the trial, as shown in experiments (Meyers et al., 2008). Overall, these results confirm our hypothesis that the differences in neural selectivity in category- vs. stimulus-specific delayed response tasks could emerge due to the task-dependent reorganization of the circuit by reward-dependent learning.

### 3.3. Multiple category boundaries

Up to now, we have looked at representations in a circuit that specializes on one specific memory task. While this scenario is useful for describing a typical behavioral experiment in monkeys, in real-life conditions the PFC needs to flexibly (and quickly) switch across a variety of different tasks.

How exactly are multiple tasks represented in PFC circuits? The answer should not come as a surprise: “it depends on the tasks.” For tasks involving non-overlapping stimuli, in particular, two independent categorization tasks (cats vs. dogs and sedans vs. sports cars), the activity of many neurons reflects both category distinctions. Thus, the neurons multitask different types of information depending on the context (Cromer et al., 2010). In contrast, when the same stimuli need to be categorized differently depending on behavioral context, the two category boundaries are represented by largely non-overlapping neuronal populations (Roy et al., 2010).

**Figure 5:**
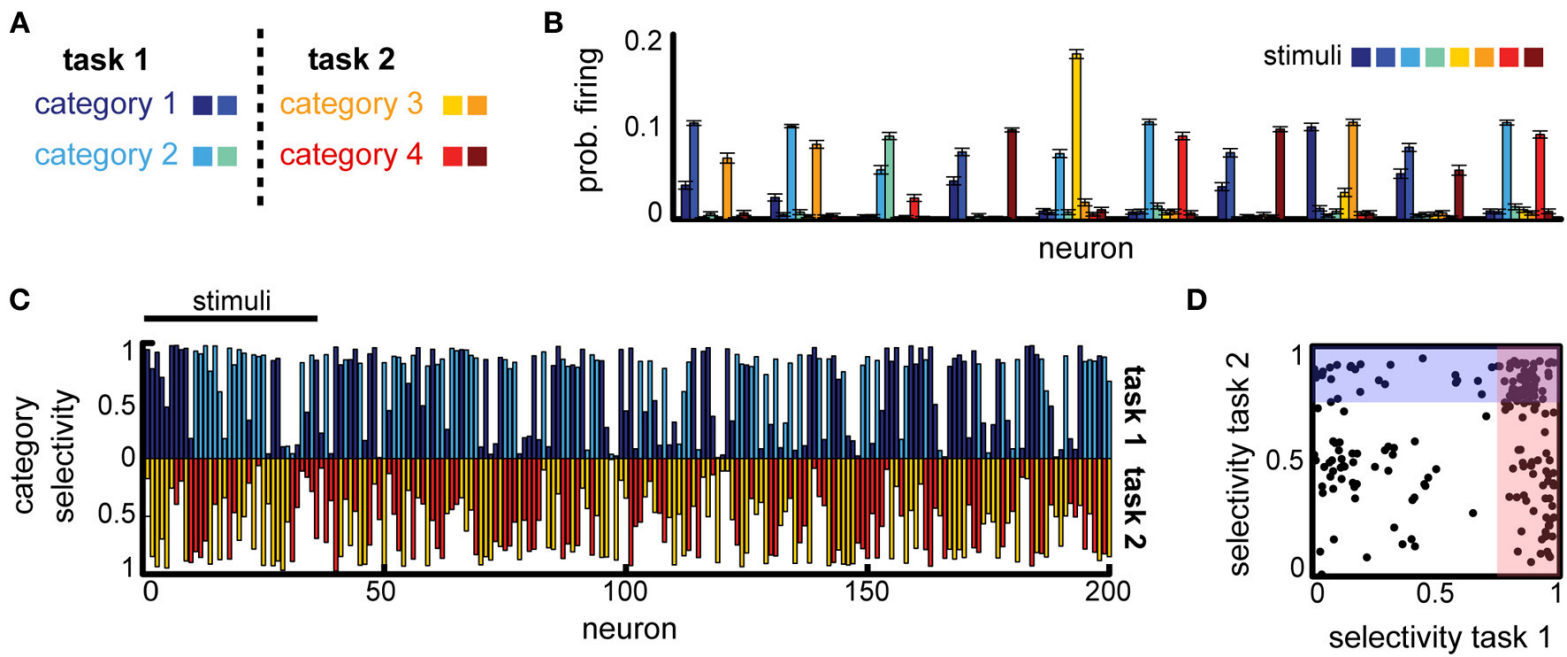
**Multitask categorization with non-overlapping domains. (A)** Given a context cue, the network needs to perform one of two categorization tasks (“task 1” or “task 2”); there are eight stimuli in total (colored squares), half of each are used in each task. **(B)** Average stimulus-specific responses for 10 randomly selected neurons. **(C)** Overlap of the category selectivity in “task 1” vs. “task 2.” **(D)** Correlation of the category specificity across tasks; shaded regions mark regions of high category selectivity.

Can a difference in task constraints explain these conflicting results? To answer this question, we constructed two versions of the multi-class delayed categorization task, similar to those used experimentally. First, to implement the multiple independent categories task we used *K* = 8 stimuli and defined two non-overlapping subsets, representing the animals and cars in the original experiment. These subsets were each split in two categories, corresponding to, e.g., the cats vs. dogs distinction, see Figure 5A, with *M* = 4 actions, corresponding to the different category distinctions. As in the basic task, the cue signal (now two inputs) was provided directly the decision layer; the cue was active for one time step at the end of the delay period. We found that the network was able to learn this task (average performance 85% for a variable delay task, with maximum delay *T*_max_ = 5). To assess the emerging neural representations learned for this task, we measured the average firing rate of the neurons in response to different stimuli. We found that many of the neurons responded strongly to several stimuli (Figure 5B). These stimuli often belonged to the same class (Figure 5B, e.g., for neurons 1, 3, 4, etc.), reproducing the category selectivity we have seen previously, but often neural responses are strong also for stimuli corresponding to different tasks (Figure 5B, e.g., the first neuron responds to category 1 and 3). Measuring the category specificity of neurons for each of the two contexts revealed that most neurons are strongly category selective (Figure 5C). Moreover, 33.5% of the neurons were sensitive to both category distinctions (selectivity threshold 0.75, see Figure 5D). This suggests that, indeed, when the tasks do not interfere with one another the circuit should multiplex information across tasks for good performance.

**Figure 6:**
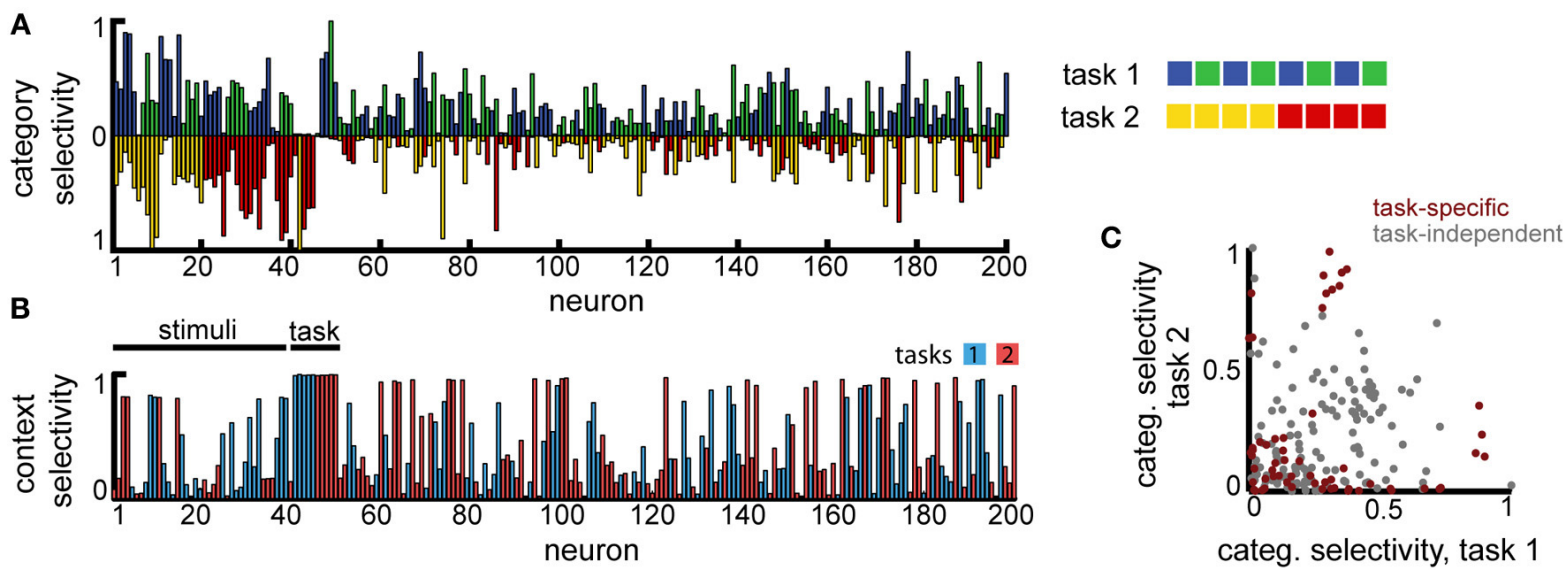
**Multitask categorization with overlapping domains**. The same eight stimuli need to be classified as belonging to class “blue” vs. “green” in task 1 or as “red” vs. “yellow” in task 2. The task to be performed in any given trial is determined by a context cue, provided as an extra input (in this example to neurons 41–50) during the initial stimulus presentation. **(A)** Overlap of the category specificity in the two tasks; color signals the preferred category for individual neurons. **(B)** The selectivity of the neuronal responses to the context; color marks the preferred context. **(C)** Correlation in category selectivity across the population. Colored dots correspond to neurons with high context selectivity (≥0.5).

Second, to model the scenario involving overlapping category boundaries, we assumed *K* = 8 input stimuli that are classified, depending on the context, using two orthogonal category boundaries (Figure 6A, right). In this case, the context needs to be provided at the beginning of the trial, together with the stimulus (the context, i.e., which task needs to be performed in the current trial, is encoded as two non-overlapping sub-populations of the same size *N*_in_, just as the stimuli). The decision layer consisted, as before, of *M* = 4 neurons, one for each category, and trials from both tasks were interleaved at random.

This version of the multiple categories experiment is significantly harder, as it requires storing information about both stimuli and the current context (because of the two extra inputs, we assumed the recurrent layer has a slightly increased firing rate *x*_0_ = 0.05). Still, the network is able to perform significantly above chance (approximatively 60%, for a variable delay with *T*_max_ = 3). In contrast to the task before, however, fewer neurons develop category specificity (19.5% as opposed to 74.5%), most represent single stimuli and several neurons encode the context itself (Figure 6B), suggesting that the network converges to a largely input-driven solution, in which information about stimuli and task is stored separately and combined only at the level of the decision layer. Among the neurons that exhibit category specificity, almost all are selective to only one of the category boundaries (points with high selectivity cluster close to the two axes, and more so if the neural responses are context modulated, see Figure 6C), unlike the previous scenario. This observation is reiterated when restricting the analysis to neurons that show task specific encoding (Figure 6C, dark red). Thus, the network organizes into separate task-specific subpopulations, as seen experimentally (Roy et al., 2010).

**Figure 7:**
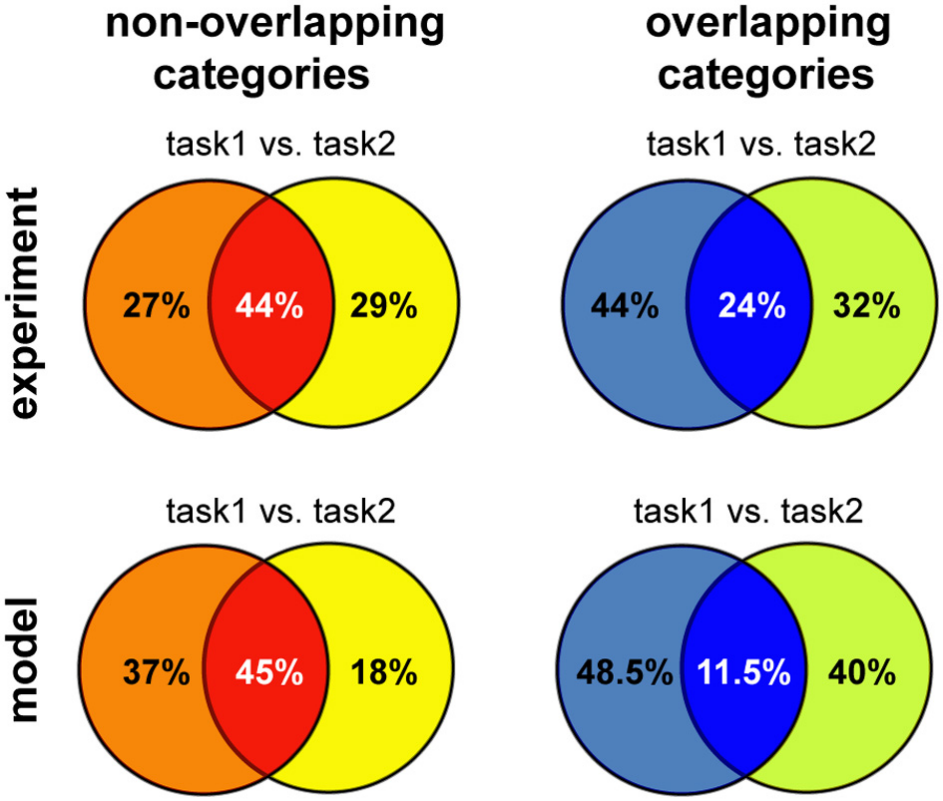
**Summary of the results for different versions of multitask categorization; comparison between model and experiments**. The category specificity at the end of learning was measured for the two variants of the multiple-category task using either overlapping or non-overlapping category boundaries. We restricted the analysis to the subset of neurons that showed any task specificity (defined as *S* ≥ *S*_specific_), as done for the experimental data analysis; the proportion of these neurons that are selective to one or both of the category boundaries was reported, averaging across five runs; *T*_max_ = 5 and *S*_specific_ = 0.75 for the non-overlapping version and *T*_max_ = 3 and *S*_specific_ = 0.5 for the overlapping categories discrimination, reflecting the increase in task difficulty. Experimental data reproduced from Cromer et al. (2010) for non-overlapping and from Roy et al. (2010) for overlapping categories, respectively.

Overall, we found that reward-dependent learning can account for the differences in category representation across experiments. Moreover, the emerging representations showed a strong task-dependent component, consistent with experimental observations (Asaad et al., 2000; Roy et al., 2010; Warden and Miller, 2010). The general match between our model and experiments is also quantitative, as shown in Figure 7 (note that the asymmetry between tasks is a consequence of a small number of experiments). This is particularly remarkable given that these results were obtained without any tuning of the model parameters, beyond that required to obtain a good performance in the simple delayed-response task. Taken together, our results suggest task demands dramatically shape neuronal representations in working-memory circuits via reward-dependent learning.

### 3.4. The importance of reward-dependent learning

To tease apart the contribution of different plasticity mechanisms to the observed effects, we compared our model to a similarly constructed network, in which weights within the recurrent layer remain fixed, or alternatively are modified by STDP independently of the obtained reward. In both cases, the readout to the decision layer was learned by r-STDP, with all homeostatic mechanisms in place.

**Figure 8:**
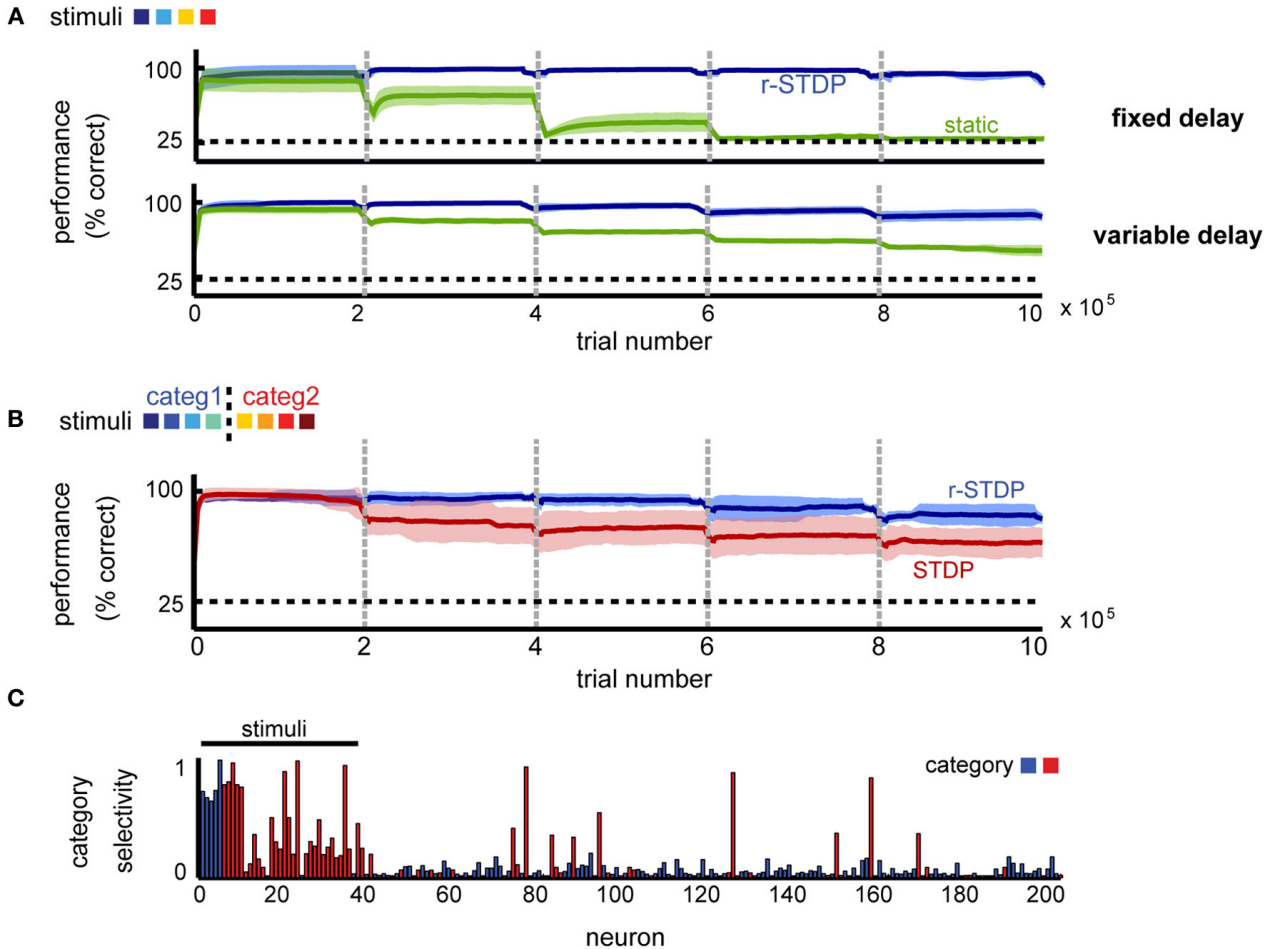
**The importance of reward-dependent learning for shaping circuit dynamics. (A)** Performance comparison of networks shaped by r-STDP (blue) vs. circuits where the recurrent connectivity is static (green) in a simple delayed response task. **(B)** Performance comparison of networks shaped by r-STDP (blue) vs. circuits where the recurrent connectivity is shaped by reward-independent STDP (red) in a 2-class categorization task; *K* = 8, fixed delay. For all conditions, the learning of the decision output was reward-modulated. Dark colors mark averages across five repetitions; light colors show the standard deviation around this mean. **(C)** Neuronal selectivity to stimulus category when learning in the recurrent circuit is reward independent; *K* = 8, fixed delay (compare to Figure 3D).

We found that learning within the recurrent layer is critical for good memory performance, and in particular that networks with r-STDP are consistently better than those in which recurrent connectivity is fixed (Figure 8A). For a simple delayed-response task (*K* = 4 stimuli), reward modulation is not strictly necessary for good performance and unsupervised learning alone can improve neural representations (the performance in unsupervised learning is indistinguishable from that using reward-dependent learning; not shown), as reported elsewhere (Lazar et al., 2009). This result is expected, since when each stimulus defines an action, it is best to represent each input as distinctly as possible, something which can be done by unsupervised learning. Indeed, the emerging representations are similar for the different learning scenarios (stimulus-specific synfire chains; not shown), such that they can be exploited for reward-dependent learning at the decision units.

Importantly, we found that simple unsupervised learning by STDP is no longer sufficient once the task difficulty is increased, by introducing more stimuli and more complex decision boundaries. Indeed, a very different picture emerges when comparing reward dependent vs. unsupervised learning in a categorization task (*K* = 8 stimuli randomly mapped into *M* = 2 categories). In this case, we find that the performance of the two differs significantly (Figure 8B). A possible reason for this difference is that attempting to represent each different stimulus separately, via unsupervised learning, exceeds the capacity of this particular network. Because if this, unsupervised learning results in poorer performance in this task. Furthermore we found that the outcome of unsupervised learning is less robust than that of reward-dependent learning: error levels depend on the particular instantiation of the network, leading to increased across trial variability (Figure 8B, shaded region in red vs. blue). This dissociation is also apparent at the level of the neural representations. After reward-independent learning, the percentage of category-specific neurons is significantly lower to both our model and the experimental data (20.5% instead of 32% for reward-dependent learning and 33% in the data; see Figure 8C). Furthermore, the network responses appear more noisy, suggesting that the number of stimuli exceed the capacity of the network and the reward-independent learning cannot learn a robust representations for all stimuli. All in all, this suggests that for complex tasks, when the pool of available resources is indeed a limiting factor, neuronal representations need to shift toward task-relevant features for good memory performance.

## 4. Discussion

Prefrontal circuits are shaped by a variety of task-related variables. These representations are likely to form during extensive training prior to experimental recordings, but the mechanisms underlying this development are poorly understood. Here, we have shown that representations similar to those reported experimentally naturally emerge in an initially unstructured circuit through reward-dependent learning. Moreover, we found that a few generic mechanisms (rSTDP and homeostasis) are sufficient to explain a range of puzzling (and seemingly complex) experimental observations. Neurons in our model developed stimulus and action specificity, both across neurons and in time, as seen experimentally (Miller et al., 1996; Chafee and Goldman-Rakic, 1998; Rainer and Miller, 2002). The same model (with no further parameter tuning) could also account for neural representations during context-dependent tasks. For tasks involving multiple independent category sets, individual neurons multiplexed information across different contexts, matching experimental observations (Cromer et al., 2010); when the same stimuli mapped into different actions depending on the context, neurons specialized to represent single category distinctions, as in Roy et al. (2010). To the best of our knowledge, our model is the first to provide an unified account of these observations.

When comparing our model to a network using reward-independent learning we found reward-dependent plasticity to be critical for solving hard tasks, such as the categorization of many stimuli. This finding is consistent with the notion that reward-dependent learning should be particularly important when resources are limited, either in terms of the amount of information that can be stored (unsupervised learning can be used to store four stimuli for the required time, but not eight), or in terms of the computations allowed for retrieving it (the readout is linear). In such scenarios, separately representing each stimulus and then mapping the neural activity into the correct output becomes unfeasible (because the resources may not suffice for representing all stimuli individually or because reading out the answer becomes too complicated). Instead, the circuit needs to compute some parts of the map between stimuli and actions during the delay, by clustering together stimuli which should yield the same behavioral response. Given generally recognized resource limitations in working memory circuits (Cowan, 2001), this finding suggests that PFC needs to be malleable, with experience shaping the sensitivity of neurons to reflect current behavior.

Here we chose a very simple model for the network dynamics, known to have small memory capacity (Büsing et al., 2010), because we wanted to focus on the recurrent circuitry and its changes during learning. It should in principle be possible to extend the memory capacity of the network closer to the biologically-relevant range (order of seconds) by using larger networks, a more realistic model of the neural dynamics and including slow time-constants, e.g., NMDA receptors (Durstewitz et al., 2000; Brunel and Wang, 2001) or short-term facilitation (Mongillo et al., 2008). Nonetheless, as the restrictions enforced by resource limitations are likely general, we expect the main features of the representations emerging in the model to be preserved, at least qualitatively, in a detailed circuit. Thus, we predict reward-dependent learning should play a general role in the formation and task-specific tuning of working memory circuits.

From a developmental perspective, it is tempting to hypothesize that reward-dependent learning may play a role in the age-dependent improvement of working memory (estimated to be approximately four-fold between the ages of 4 and 14) (Luciana and Nelson, 1998), complementing other known factors such as the maturation of the underlying cortical architecture, a better representation of the inputs, the development of attention, or the usage of memorization strategies such as rehearsal and chunking (Gathercole, 1999). This suggestion is consistent with the known dependence of PFC function on dopamine in early life (Diamond and Baddeley, 1996). Furthermore, the same mechanisms may account for training-induced improvements in working memory in adults (Klingberg, 2010).

From a broader computational perspective, our work is also relevant in the context of reservoir computing (Lukoševičius and Jaeger, 2009). While this framework traditionally assumes fixed recurrent connectivity, recent work has increasingly argued for the importance of learning in shaping reservoir properties (Schmidhuber et al., 2007; Haeusler et al., 2009; Lazar et al., 2009). Previous work used general-purpose optimization through unsupervised learning. Here, however, the network is shaped directly by the task, which improves performance significantly compared to static networks or networks shaped by reward-independent learning. Thus, our model provides a stepping stone toward general task-specific optimization of recurrent networks.

Time-dependent representations are preferred to traditional attractor-based solutions (Amit and Brunel, 1997; Brunel and Wang, 2001; Mongillo et al., 2008) in our model, consistent with recent experimental observations (Miller et al., 1996; Chafee and Goldman-Rakic, 1998; Pesaran et al., 2002; Rainer and Miller, 2002; Barak et al., 2010) and previous theoretical predictions (Goldman, 2009). This effect is a consequence of intrinsic plasticity, which discourages neurons from remaining active for a long time (Horn and Usher, 1989). Given that homeostasis plays a critical role in stabilizing the circuit dynamics during learning (Turrigiano and Nelson, 2004), the fact that the emerging representation is time-varying is not really surprising. While our model emphasizes the temporal component of this representation, it is likely that the patterns of activity seen experimentally emerge through the interaction between feedforward and feedback dynamics, which would require a more detailed model of the neural dynamics. Although the homeostatic mechanisms acting in PFC circuits have yet to be characterized experimentally, it is tempting to assume that the sparsification of activity and increase in robustness observed experimentally after training (Rainer and Miller, 2000) may be signatures of the interaction between hebbian and homeostatic plasticity as shown in our model. More generally, similar mechanisms could play a role in developing feedforward dynamics in other recurrent circuits (see also Levy et al., 2001; Buonomano, 2005; Gilson et al., 2009; Fiete et al., 2010), for instance in other areas known to exhibit delay period responses, such as the perirhinal cortex, inferotemporal cortex, or the hippocampus (Miller et al., 1993; Quintana and Fuster, 1999).

Our model combines both hebbian (r-STDP) and homeostatic (intrinsic plasticity, synaptic scaling) forms of plasticity, lending further support to the notion that the interaction between different forms of plasticity is critical for circuit computation (Triesch, 2007; Lazar et al., 2009; Savin et al., 2010). In particular, our results confirm the computational importance of intrinsic plasticity and synaptic scaling in excitatory neurons (Savin et al., 2010; Keck et al., 2012). To this, we add the role of inhibitory plasticity, which we found improved both neural representations and memory performance.

We view this model as a starting point for investigating reward-dependent learning in working memory circuits, to which many additions can be made. While the abstract network model used here allowed us to focus on the essential mechanisms underlying PFC coding, it would be important to investigate reward-dependent learning in more realistic spiking neural networks. Furthermore, the model for different plasticity mechanisms operating in the network could be refined as well. First, reward-dependent learning could be improved by using recent extensions of r-STDP to spiking neuron populations (Urbanczik and Senn, 2009). Second, the simplistic regulation of inhibition should be replaced by realistic inhibitory plasticity (Castillo et al., 2011), which is expected to also aid network selectivity (Vogels et al., 2011). Third, activity-dependent structural plasticity could optimize the cortical connectivity to best encode the task-specific information (Savin and Triesch, 2010; Bourjaily and Miller, 2011), consistent with experimental observations that working memory training alters circuit connectivity (Takeuchi et al., 2010). Lastly, preliminary work, supported by recent observations about the effects of neuromodulation on inhibitory and homeostatic plasticity (Seamans et al., 2001; Di Pietro and Seamans, 2011), suggests that the homeostatic plasticity mechanisms themselves may be reward-dependent.

## Author contributions

Designed the experiments: Cristina Savin and Jochen Triesch; Implemented the model and analyzed the data: Cristina Savin; Wrote the paper: Cristina Savin and Jochen Triesch.

### Conflict of interest statement

The authors declare that the research was conducted in the absence of any commercial or financial relationships that could be construed as a potential conflict of interest.
